# Rethinking the Bland-Altman plot when quantifying virtual coronary physiology

**DOI:** 10.3389/fcvm.2025.1687923

**Published:** 2025-10-23

**Authors:** Daniel J. Taylor, Harry Saxton, Ian Halliday, Julian P. Gunn, Paul D. Morris

**Affiliations:** ^1^ Division of Clinical Medicine, School of Medicine and Population Health, University of Sheffield, Sheffield, United Kingdom; ^2^ Insigneo Institute for in Silico Medicine, University of Sheffield, Sheffield, United Kingdom; ^3^ NIHR Sheffield Biomedical Research Centre, Sheffield Teaching Hospitals NHS Foundation Trust, Sheffield, United Kingdom; ^4^ School of Computer Science, University of Sheffield, Sheffield, United Kingdom; ^5^ Department of Cardiology and Cardiothoracic Surgery, Sheffield Teaching Hospitals NHS Trust, Sheffield, United Kingdom

**Keywords:** coronary artery disease, FFR, virtual physiology, Bland-Altman, agreement

## Abstract

Angiography-derived, or “virtual” fractional flow reserve (vFFR) is beginning to replace invasive assessment in some catheterisation laboratories. Despite its incorporation into clinical guidelines, recent clinical outcomes data have cast doubt over its effectiveness relative to invasive assessment. These somewhat unexpected trial results are underpinned by poorer than anticipated agreement between invasive and vFFR. In particular, the widespread use of traditional Bland-Altman analysis fails to account for the phenomenon of worsening agreement at lower FFR values which hinders comparison between studies. We propose a novel approach using quantile regression to derive overall bias and limits of agreement (LOA) to better characterise agreement across the spectrum of coronary disease. This new method may improve understanding of optimal vFFR clinical applications and addresses common statistical deficiencies in current validation practices.

## 1 Introduction

Virtual fractional flow reserve (vFFR), derived from standard coronary angiography, was first described over ten years ago (1) and offers improved decision-making with reduced procedural burden. Since its conception, several validation trials (2, 3) have provided a body of evidence supporting its ability to predict invasive FFR. These data supported the recent endorsement for its use in the assessment of chronic coronary syndromes (CCS) in the European guidelines (4). For most cases seen in everyday clinical practice, where the significance of lesions is judged from angiography alone, vFFR-guided therapy is superior to angiography for major adverse cardiac events (MACE) (5). However, recent trial outcome data have challenged assumptions about the equivalence of vFFR when compared to the gold-standard physiological assessment – invasive FFR (6). Failure of vFFR-guided therapy to meet non-inferiority for MACE likely resulted from poorer than anticipated agreement between invasive FFR and vFFR. This indicates discrepancies between the techniques which are not necessarily obvious from published statistical analyses. This real-world mismatch may result from worsening agreement at decreasing FFR (grey zone and lower values). Consequently, studies reporting agreement in a cohort containing more non-haemodynamically significant lesions, than would be subject to vFFR assessment in everyday clinical practice, may produce misleading results.

**Figure 1:**
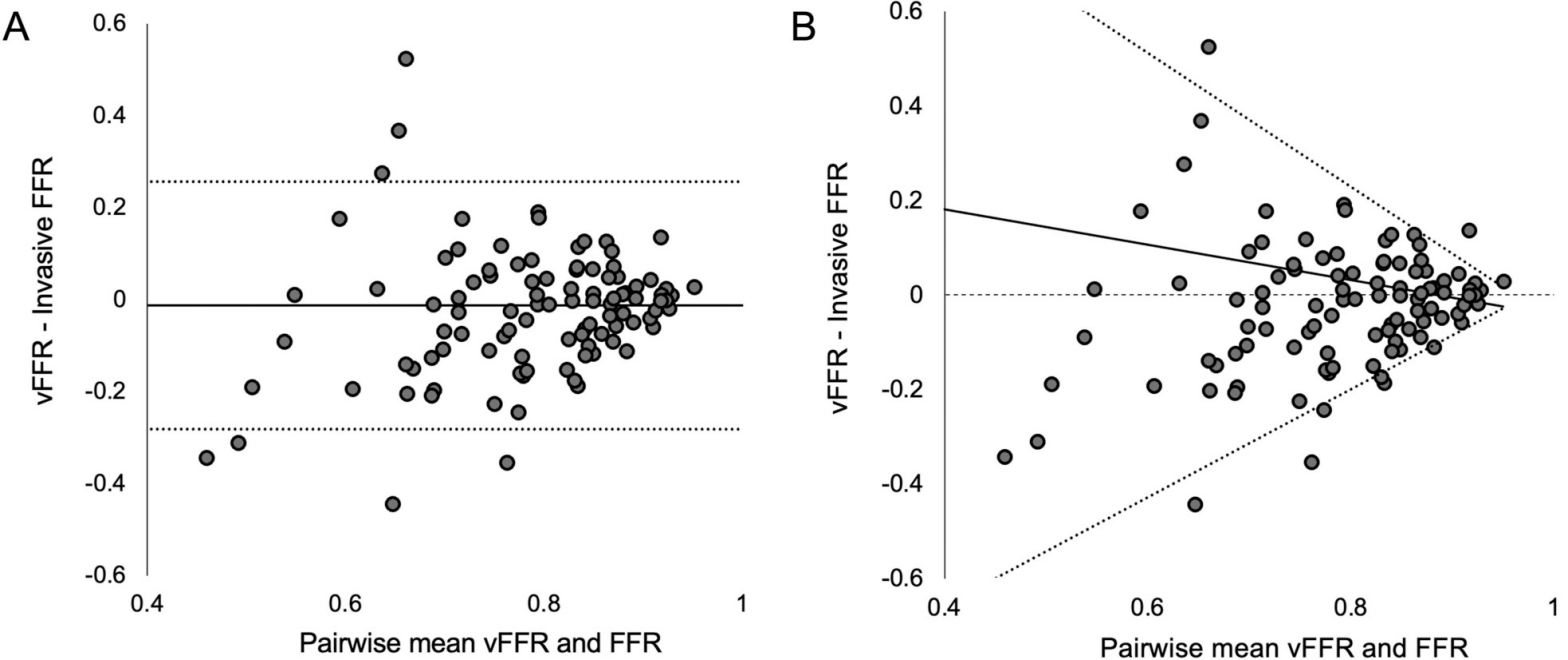
Standard Bland-Altman plot and plot with quantile-derived median bias and limits of agreement. Panel **(A)** Standard Bland-Altman plot with mean bias −0.01 (95% LOA −0.28 to 0.26). Panel **(B)** Quantile-derived LOA same data fitted with quantile regression, at pairwise mean (FFR + vFFR)/2 value of 0.80, median bias was +0.03 (95% LOA −0.20 to 0.23). At pairwise mean (FFR + vFFR)/2 value of 0.70, median bias was +0.07 (95% LOA −0.31 to 0.37) and at 0.90 median bias was −0.01 (95% LOA −0.09 to 0.09). Solid line represents overall bias, dashed lines represent 95% LOA. Note the widening LOA at lower pairwise mean (FFR + vFFR)/2 values, consistent with greater variability in agreement at higher disease severity. LOA, limits of agreement; FFR, fractional flow reserve; vFFR, virtual Fractional Flow Reserve.

The Bland-Altman plot, first introduced in 1986, remains the gold standard for assessing agreement between two clinical measurement techniques (7). The technique was devised as an easy-to-use tool, specifically targeted at clinical applications, which moves beyond simple regression and correlation. This parametric technique plots the average of two measurements against the difference between them. On the plot, the mean delta gives the overall bias and the 95% limits of agreement (LOA) represent the range within which 95% of the differences between two measurement methods are expected to lie. Both are plotted (Figure 1). The plot therefore gives a more visually intuitive presentation of the data vs. a simple scatter plot. It has proved extremely popular and is now one of the most highly cited statistical manuscripts in existence (over 53,000 at the time of writing). It is widely used in vFFR validation studies (1, 8, 9). However, key assumptions of the Bland-Altman method — including homoscedasticity (equal variance across measurement range) — are routinely violated in vFFR validation datasets, where measurement error frequently varies with disease severity. This is certainly not exclusive to vFFR analyses but is well demonstrated by their application. This results in traditional LOA calculations underestimating accuracy in healthier vessels while overestimating it in more diseased arteries. As a result, true agreement between vFFR and FFR remains poorly understood. While statistical workarounds such as transformation of data may provide a solution (10, 11), these detract from a key principle of the Bland-Altman plots – simplicity and ease of access for those without a more thorough statistical grounding. The lack of, to our knowledge, any vFFR validation study employing these alternative techniques to ensure an appropriate fit of data suggests they do not meet these criteria.

In this Perspective, we propose a refined statistical framework using quantile regression to generate more appropriate LOA, enhancing the clinical interpretation of vFFR performance across a spectrum of disease severities. Crucially, this technique is easily implemented and understood for those familiar with the original Bland-Altman technique.

## 2 Why vFFR agreement is unsuited to traditional Bland-Altman assessment

The Bland-Altman plot displays the difference between two paired measurements against the pairwise mean of measurements. The mean bias is then plotted, along with the 95% LOA defining the expected spread of differences (Figure 1). This parametric test is dependent upon two key assumptions: differences must be normally distributed and the variance (spread) of differences should be constant across the range of measurement (homoscedasticity). These assumptions are rarely satisfied for vFFR. Principally, as vFFR quantifies a ratio between 0 and 1, results close to the higher of these values are limited in the maximum possible error. For example, a case producing an average FFR and vFFR result of 0.95 may only have a maximum error of 0.10 (results of 0.90 and 1.00), yet this phenomenon is entirely lost when LOA typically exceed ±0.10. Furthermore, accuracy is inherently dependent upon stenosis severity. In mild disease, anatomical and physiological surrogacy is stronger, resulting in tighter agreement. In contrast, in severe disease or borderline cases, vFFR models are prone to larger error, often due to greater lesion severity or complexity, leading to broader discrepancies. Despite this, Bland-Altman analysis is important for evaluating vFFR performance across studies. When applied correctly, its results are more robust to case selection compared with diagnostic accuracy metrics such as sensitivity and specificity, therefore facilitating inter-study comparison.

## 3 A new proposal: quantile regression-derived limits of agreement

We propose an enhancement to the traditional Bland-Altman approach by calculating overall bias and LOA using quantile regression (12). Instead of assuming constant variance, quantile regression models the spread of differences across the range of measurements, generating dynamic LOA that expand or contract in a manner which is more likely to appropriately model the typical variations in vFFR studies. A key reason we advocate for this approach reflects an underpinning value of the original Bland-Altman plot – simplicity. Our approach can be easily implemented with basic statistical software (for our study we used the quantreg package in R version 2024.04.2 + 764) and does not require more advanced statistical knowledge about data transformation. The technique could be almost universally applied to vFFR validation studies; even if the data unexpectedly met the assumptions required for traditional Bland-Altman analysis, the effect on overall bias and LOA would be minimal. A key distinction to make, is while the original Bland-Altman reports **mean** bias, quantile regression reports **median** bias which is more appropriate for non-parametric data. An example application is shown in Figure 1 with data taken from a recent validation study conducted by our group (13). Panel A shows the traditional Bland-Altman plot with fixed LOA, while Panel B demonstrates our proposed method using quantile regression. The divergence of LOA with increasing disease severity is readily apparent, accurately capturing worsening measurement variability as FFR values decrease. To aid comparison, we propose reporting the median bias and 95% LOA principally at the pairwise average (FFR + vFFR)/2 value of 0.80 (the diagnostic threshold). Authors may also wish to report LOA at 0.70 and 0.90, to quantify the relationship between agreement with disease severity.

## 4 Discussion

In this article, we have described a simple method of assessing agreement between invasive and vFFR which accounts for commonly encountered issues with data distribution. Our method directly addresses the heteroscedasticity inherent to coronary physiology and virtual modelling. Specifically, the technique considers the phenomenon of poorer agreement with more diseased cases in a visually intuitive way and may also be more appropriate for assessing microvascular resistance (14, 15). vFFR is now a guideline indicated tool in the assessment of intermediate coronary artery stenoses. While evidence supports it's superiority vs. standard angiographic assessment (5), it does not meet non-inferiority vs. invasive physiology (6). This difference is underpinned by agreement with invasive physiology, but traditional Bland-Altman analysis does not fully capture this relationship. While the proposed technique does not always guarantee an optimal data fit, and other non-parametric approaches may be used when fitting LOA to non-parametric data (such as with polynomials) (16), the simplicity in implementing, interpreting and comparing results strikes a balance which also preserves the familiarity of the original Bland-Altman plot. We therefore encourage other authors to consider this statistical technique when performing their own vFFR validation studies.

## Data Availability

Data underlying this work are available upon reasonable request to the corresponding author.
